# Food-borne botulism outbreak during the Rugby World Cup linked to marinated sardines in Bordeaux, France, September 2023

**DOI:** 10.2807/1560-7917.ES.2023.28.41.2300513

**Published:** 2023-10-12

**Authors:** Léa Courtot-Melciolle, Marine Jauvain, Mona Siefridt, Renaud Prevel, Olivia Peuchant, Olivier Guisset, Gaëlle Mourissoux, Laure Diancourt, Christelle Mazuet, Gauthier Delvallez, Alexandre Boyer, Arthur Orieux

**Affiliations:** 1 Medical Intensive Care Unit, CHU Bordeaux, Bordeaux, France; 2 Bacteriology Department, CHU Bordeaux, Bordeaux, France; 3 INSERM UMR 1045, Centre de Recherche Cardio-Thoracique de Bordeaux, Bordeaux University, Bordeaux, France; 4 National Reference Center for Anaerobic Bacteria and Botulism, Institut Pasteur, Université Paris Cité, Paris, France

**Keywords:** outbreak, food-borne botulism, intensive care unit, invasive mechanical ventilation, health authorities

## Abstract

In September 2023, a botulism outbreak affecting 15 individuals occurred in Bordeaux, France, during the Rugby World Cup. We report on eight individuals from four different countries on two continents admitted to the intensive care unit at our hospital, where six required invasive mechanical ventilation. Cases reported consuming locally produced canned sardines at a restaurant. This report highlights the importance of rapid, worldwide alerts from health authorities to prevent severe consequences of such outbreaks, particularly during events attracting international visitors.

An outbreak of 15 cases (including one death) of food-borne botulism occurred in Bordeaux, France, in September 2023 during the Rugby World Cup. Here, we present the clinical case descriptions of the eight individuals treated at Bordeaux University Hospital, the laboratory identification of type B botulinum neurotoxin (BoNT) and the control measures implemented to stop the outbreak.

## Case descriptions

On 6 September 2023, the first patient was admitted to the medical intensive care unit (ICU) at our hospital. The patient presented with bilateral oculomotor palsy, mydriasis, ptosis, impaired wallowing, nausea and vomiting, and required invasive mechanical ventilation. Because of the neurological symptoms, the patient was initially treated for Guillain–Barré syndrome, but botulism was also suspected. On 9 and 10 September 2023, two additional patients were admitted to the medical ICU with similar neuro-ophthalmic, digestive, ear, nose and throat symptoms. All three patients were visiting France to attend the rugby tournament or tourism.

The public health authorities were contacted when an outbreak was suspected, on 10 September. Patient histories revealed that the suspected source of infection was home-canned sardines consumed in the same bar/restaurant in Bordeaux by all three individuals [1]. 

**Figure 1:**
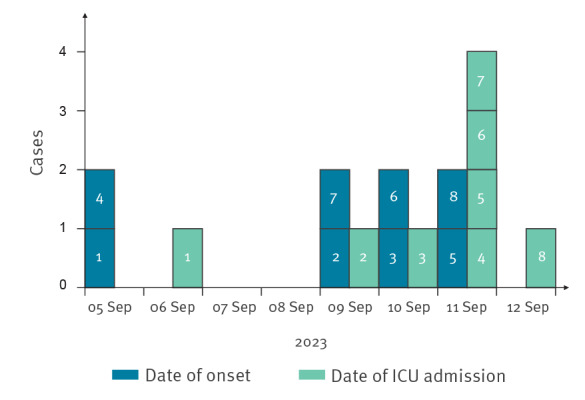
Botulism cases by date of symptom onset and intensive care unit admission, Bordeaux University Hospital, Bordeaux, France, September 2023 (n = 8)

On 11–12 September 2023, five additional patients, also international visitors in France, were hospitalised at our hospital with clinical signs of botulism (Table) (Figure 1). 

**Table:** Description of botulism cases hospitalised at Bordeaux University Hospital, Bordeaux, France, September 2023 (n = 8)

Case	Symptoms					Clinical outcomes			Laboratory testing			
									Serum sample^a^		Rectal or stool sample^b^	
	Neuro-ophthalmic	Digestive	ENT	Other	Time to onset	ICU admission	Orotracheal intubation	Antitoxin administration	Date	Result	Date (sample)	Result
1	Oculomotor palsy, mydriasis, ptosis	Nausea, vomiting	Impaired swallowing	None	15 h	6 Sep, 13:00	6 Sep, 15:00	12 Sep, 08:00	7 Sep, 09:30	Positive (type B BoNT)	11 Sep, 06:00 (rectal swab)	Negative
2	Oculomotor palsy, mydriasis, ptosis	Nausea, vomiting	Impaired swallowing	None	11 h	9 Sep, 21:00	9 Sep, 22:00	11 Sep, 12:00	11 Sep, 06:40	Strong suspicion of BoNT	9 Sep, 22:30 (stool sample)	Positive (type B Cb)
3	Oculomotor palsy, mydriasis, ptosis	None	Impaired swallowing, dysphonia, dysarthria	Headache	13 h	10 Sep, 21:00	11 Sep, 10:00	11 Sep, 21:00	11 Sep, 05:00	Strong suspicion of BoNT	11 Sep, 23:00 (rectal swab)	Negative
4	Ptosis	Nausea, diarrhoea	Dysphagia	Descending paralysis, chest pain	13 h	11 Sep, 11:00	None	12 Sep, 01:00	11 Sep, 11:40	Negative	11 Sep, 16:00 (rectal swab)	Negative
5	Oculomotor palsy, mydriasis, blurry vision, ptosis	Nausea, vomiting	Impaired swallowing	Descending paralysis, respiratory distress	59 h	11 Sep, 12:00	11 Sep, 19:00	12 Sep, 08:00	12 Sep, 12:00	Positive (type B BoNT)	12 Sep, 12:00 (rectal swab)	Positive (type B Cb)
6	Oculomotor palsy, mydriasis	Diarrhoea	Dysphagia, dysphonia, dysarthria	None	39 h	11 Sep, 13:00	None	12 Sep, 8:00	11 Sep, 16:20	Negative	13 Sep, 13:00 (rectal swab)	Negative
7	Oculomotor palsy	Diarrhoea	Impaired swallowing, dysphonia, dysarthria	None	11 h	11 Sep, 15:00	13 Sep, 13:00	12 Sep, 11:00	11 Sep, 16:15	Negative	13 Sep, 11:00 (rectal swab)	Positive (type B Cb)
8	Oculomotor palsy, mydriasis, blurry vision, ptosis	Nausea, vomiting	Impaired swallowing, dysphonia, dysarthria	None	18 h	12 Sep, 02:30	12 Sep, 16:00	12 Sep, 12:00	12 Sep, 02:30	Strong suspicion of BoNT	12 Sep, 02:30 (stool sample)	Positive (type B Cb)

BoNT: botulinum neurotoxin; Cb: *Clostridium botulinum*; ENT: ear, nose, throat; ICU: intensive care unit.

^a^ Detection of botulinum neurotoxin (BoNT) was confirmed using a mouse bioassay (intraperitoneal administration of patient serum to mice) [2]. BoNT serotype was determined by neutralisation of toxicity in mice by serotype-specific antibodies. Strong suspicion of BoNT indicates that patient sera caused toxic activity in the mouse bioassay, but positivity could not be confirmed by seroneutralisation.

^b^ Detection and characterisation of *Clostridium botulinum* was performed by real-time PCR targeting BoNT-producing clostridia [3], with enrichment culture on stool samples or rectal swabs.

ICU: intensive care unit.

Numbers 1–8 correspond to the eight cases described in the Table.

**Figure 2:**
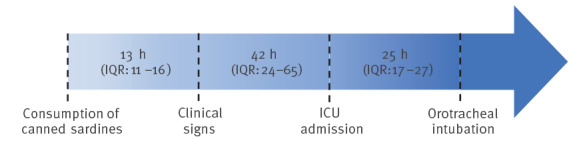
Temporal representation of botulinum toxin infection and course of treatment in cases hospitalised at Bordeaux University Hospital, Bordeaux, France, September 2023 (n = 8)

ICU: intensive care unit; IQR: interquartile range.

Overall, eight cases were treated at the medical ICU; they were from Canada, France, Ireland and the United States, two were male, six were female and seven of eight cases were under 50 years old; one had an underlying neurological condition. The median delay between consumption of sardines and symptom onset was 13 h (interquartile range (IQR): 11–16) (Figure 2). Patients were admitted to the ICU with a median delay of 42 h (IQR: 24–65) after the onset of symptoms. Six of eight cases required invasive mechanical ventilation because of respiratory muscle paralysis. The median delay between onset of symptoms and orotracheal intubation of 25 h (IQR: 17–27) hours. 

As on 12 October 2023, the median time of hospitalisation was 18 days (IQR: 8–21) and two patients still required invasive mechanical ventilation.

All eight patients were treated with botulism antitoxin (Botulism Antitoxin Heptavalent, Emergent BioSolutions, Canada). By the date of publication (12 October 2023), six of eight patients had been discharged, and two patients still required invasive mechanical ventilation.

## Laboratory confirmation of botulism

Before administration of antitoxins, sera from the eight hospitalised cases were collected (Table) and sent to the French National Reference Center (NRC) for Anaerobic Bacteria and Botulism to confirm detection of botulinum neurotoxin (BoNT) using a mouse bioassay (intraperitoneal inoculation of patient serum) [2]. The BoNT serotype was determined by neutralisation of toxicity in the mice by serotype-specific antibodies. The presence of BoNT type B was confirmed in two cases (Table), as serum injection induced death in the mice within 24 h. The sera from three other cases caused toxic activity in the bioassay; the mice showed respiratory distress, abdominal girdle shrinkage, and posterior traction motor paralysis, strongly suggestive of BoNT, but this could not be confirmed by seroneutralisation. The amount of free toxin in these sera was probably lower and close to the detection limit of this test. For the three remaining cases, the mouse bioassay test did not detect BoNT.

In addition, detection and characterisation of the bacteria was performed by real-time PCR targeting BoNT-producing clostridia [3], with enrichment culture on stool samples or rectal swabs. The majority of cases (6/8) were constipated, and when stools could not be obtained, a rectal swab was taken for testing. *Clostridium botulinum* type B was detected in four cases, confirming microbiological diagnosis of botulism for two additional cases.

In total, five cases were confirmed, two with the mouse bioassay (serum sample, Cases 1 and 5) and three additional by real-time PCR targeting BoNT-producing clostridia (stool samples or rectal swabs, Cases 2, 7 and 8). Three patients (suspected cases, Cases 3, 4 and 6) did not have biological confirmation. All laboratory tests were negative for the two cases who did not require invasive mechanical ventilation. Of note, some of the rectal swabs were extremely low in faecal material, which may explain why *C. botulinum* was not detected.

The suspected food (sardines) from the restaurant was also analysed by real-time qPCR at the NRC on 13 September. Results confirmed the presence of BoNT and type B *C. botulinum*. 

## Public health control measures

The French National Public Health Agency was notified of the three suspected cases of food-borne botulism on 11 September 2023. The same day, measures were implemented by the Departmental Directorate for the Protection of Populations of Gironde (DDPP) in the restaurant to stop the outbreak, consisting of removal of the suspected food and subsequent bacteriological [1]. The investigation ascertained that the sardines were prepared and served only at the restaurant, and not distributed further. On 12 September 2023, the French Directorate General of Health (DGS) sent a national alert to all practitioners and reported 10 cases, including eight hospitalisations at the Bordeaux University Hospital ICU and one death linked to this outbreak [4].

On 13 September 2023, the French National Public Health Agency issued an online press release recommending that people who had visited the restaurant between 4 and 10 September should contact medical practitioners in case of symptoms compatible with botulism [5]. The European Centre for Disease Prevention and Control (ECDC) was contacted by the French public health authorities on 13 September to assess the possible risk outside France [6]. On 14 September, the French health authorities notified the World Health Organization (WHO) [7].

## Discussion

Botulism is a rare and potentially severe neuroparalytic disease caused by BoNTs, mainly produced by the bacterium *C. botulinum* [8]. In 2021, 82 confirmed cases of botulism were reported in the European Union [9]. Food-borne botulism is the most common form of the disease and usually caused by inadequately processed, often home-canned, preserved or fermented foods. In France, during the 2008–18 period, 82 outbreaks of food-borne botulism were reported, representing a total of 159 cases, and the maximum number of people involved in a single outbreak was six [10,11].

Bordeaux was one of the host cities for the Rugby World Cup 2023, held throughout France from 8 September to 28 October; the tournament welcomed thousands of international visitors. According to a report by the French National Public Health Agency, this food-borne botulism outbreak, which affected 15 individuals in total, affected individuals from different nationalities (Canada, France, Germany, Ireland, Spain, United Kingdom and United States)[4]. All 15 reported eating sardines at the same restaurant [4]. We report data from the eight patients admitted to our hospital. 

*Clostridium botulinum* of type B was identified, which is the most common serotype in human botulism cases in the European Union/European Economic Area (EU/EEA) [9]. Several toxin serotypes can cause disease in humans, and type B toxin infection is usually linked to ingestion of canned foods [12]. However, toxin B is less indicative of a food-borne outbreak linked to fish consumption than toxin E. It is possible that the botulism outbreak in Bordeaux, caused by the BoNT/B toxin, could be linked to the use of olive oil and aromatic herbs (marinade) [13] before canning sterilisation of the sardines.

Botulism is commonly diagnosed by observing clinical signs and symptoms that are consistent with the illness. Nevertheless, the neurological symptoms and the progression sequence are sometimes misdiagnosed [14,15]. Indeed, some neurological diseases, e.g. myasthenia gravis and Guillain–Barré syndrome, have signs and symptoms that overlap with botulism, which may result in initial misdiagnosis [16]. Most patients have difficulty swallowing, blurry vision, slurred speech, mydriasis and descending flaccid paralysis. Paralysis involves respiratory muscles in most instances, requiring admission to the ICU for ventilatory support. Thus, the initial management for patients with suspected botulism must be made based on clinical findings. Testing of blood, stool and suspected food sources can confirm the diagnosis, but these results may not be available for several days and treatment should not be delayed.

Botulism is often responsible for severe disease with prolonged hospitalisation and mechanical ventilation. Given the development of ventilator support in the ICU, botulism is rarely lethal today, even if respiratory failure at the acute phase or ICU complications later in the course of disease can still cause death. One individual who visited the emergency unit of our hospital with atypical symptoms (pharyngitis) during the outbreak period died a few days thereafter in Ile de France. All patients treated at our hospital were treated with botulism antitoxin with a median time between ICU admission and antitoxin administration of 19 h (IQR: 13–32). At day 30, six of eight patients had been discharged, and two patients still required invasive mechanical ventilation. These elements underline the importance of a rapid and widespread health alert. Treatment is mainly supportive, with the addition of specific medications such as antitoxins [17,18]. When administered early (within 24–48 h from the onset of symptoms), botulinum antitoxin could stop the progression of paralysis and prevent respiratory failure [19]. 

## Conclusion

Botulism is a relatively rare disease in France and in Europe, but cases continue to occur sporadically. Food-borne botulism can be misdiagnosed. This report highlights the importance of promptly notifying cases with suspected botulism, as this triggers awareness and immediate investigation to determine the source and control the outbreak, but also rapid identification of others potentially linked to the outbreak so appropriate medical treatment can be given.
