# Author’s correction for Euro Surveill. 2023;28(41)

**DOI:** 10.2807/1560-7917.ES.2023.28.43.231026c

**Published:** 2023-10-26

**Authors:** Eurosurveillance editorial team

**Affiliations:** 1 European Centre for Disease Prevention and Control (ECDC), Stockholm, Sweden

In the article entitled ‘Food-borne botulism outbreak during the Rugby World Cup linked to marinated sardines in Bordeaux, France, September 2023’ by Courtot-Melciolle et al. published on 12 October 2023, the date that the French National Public Health Agency was notified of the three suspected cases of food-borne botulism was reported as 11 September 2023. The correct date for notification was on 10 September in the evening. This was corrected on 23 October 2023 at the request of the authors.

